# Photocatalytic methane oxidation over a TiO_2_/SiNWs p–n junction catalyst at room temperature

**DOI:** 10.3762/bjnano.15.92

**Published:** 2024-09-02

**Authors:** Qui Thanh Hoai Ta, Luan Minh Nguyen, Ngoc Hoi Nguyen, Phan Khanh Thinh Nguyen, Dai Hai Nguyen

**Affiliations:** 1 Institute of Chemical Technology, Vietnam Academy of Science and Technology, 1A TL29 Street, Thanh Loc Ward, District 12, Ho Chi Minh City 700000, Vietnam; 2 Graduate University of Science and Technology, Vietnam Academy of Science and Technology, 18 Hoang Quoc Viet Street, Cau Giay District, Hanoi 100000, Vietnam; 3 Department of Chemical and Biological Engineering, Gachon University, 1342 Seongnamdaero, Sujeong-gu, Seongnam-si, Gyeonggi-do 13120, Republic of Korea

**Keywords:** photocatalysis, photocatalytic CH_4_ oxidation, p–n heterojunction, TiO_2_/SiNWs

## Abstract

Rapid recombination of charge carriers in semiconductors is a main drawback for photocatalytic oxidative coupling of methane (OCM) reactions. Herein, we propose a novel catalyst by developing a p–n junction titania–silicon nanowires (TiO_2_/SiNWs) heterostructure. The structure is fabricated by atomic layer deposition of TiO_2_ on p-type SiNWs. The TiO_2_/SiNWs heterostructure exhibited an outstanding OCM performance under simulated solar light irradiation compared to the single components. This enhanced efficiency was attributed to the intrinsic electrical field formed between n-type TiO_2_ and p-type SiNWs, which forces generated charge carriers to move in opposite directions and suppresses charge recombination. Besides, surface morphology and optical properties of the the p–n TiO_2_/SiNWs catalyst are also beneficial for the photocatalytic activity. It is expected that the results of this study will provide massive guidance in synthesizing an efficient photocatalyst for CH_4_ conversion under mild conditions.

## Introduction

Methane (CH_4_), which can take the form of liquefied natural gas, is one of the crucial sources of industrial chemicals and energy. However, CH_4_ is a major precursor for tropospheric ozone, causing severe air pollution. Because of its rising atmospheric concentration, CH_4_ poses a global warming potential approximately thirty fold larger than that of carbon dioxide (CO_2_) [1–3]. Therefore, it is a challenging mission to eliminate CH_4_ from the atmosphere. Conventionally, CH_4_ activation is carried out at high temperatures (>650 °C) via thermal methane conversion to value-added products. However, combustion of CH_4_ for energy production usually generates great amounts of carbon dioxide as well as coke deposition on catalyst surfaces [4–7]. Therefore, sustainable strategies for both green conversion and atmospheric removal of CH_4_ are urgently necessary [8–11]. Semiconductor-based photocatalysis has been attracting scientists’ attention because of its environmental friendliness and easy handling [12–14]. Photocatalytic metal oxide semiconductor materials have been utilized for converting solar energy into valuable chemical energy in the field of CH_4_ conversion [15–17]. Methane oxidation presents a particularly promising strategy. The primary objective is to convert methane into valuable products such as formaldehyde (HCHO), methanol (CH_3_OH), and other value-added oxygenates, which serve as essential precursors in various manufacturing and production processes [18–19].

The n-type semiconductor titanium dioxide (TiO_2_) has been discovered as a potential photocatalyst material because of its high stability, good dispersibility, and narrow energy bandgap. However, pristine TiO_2_ shows only low photocatalytic efficiency because of the high recombination rate between holes and electrons and the low visible-light harvesting ability [20–22]. The rapid recombination of charge carriers prior to their participation in reactions significantly reduces the efficiency of methane oxidation reactions [23–24]. To address these issues of TiO_2_ nanomaterials, many scientists have developed TiO_2_-based nanostructure composites as advanced photocatalysts [25–30]. The recombination of charge carriers is mainly attributed to the anisotropic movement of generated electron–hole pairs in semiconductors. Therefore, the implementation of a driving force could remarkably accelerate the oriented motion of electrons and holes, which could suppress recombination and eventually improve photocatalytic efficiency. For years, doping of metal nanoparticles (NPs) into a semiconductor matrix has been extensively studied to enhance photocatalytic CH_4_ oxidation performance. Metal NPs in, for example, Au/TiO_2_, Au@Pd/ZnO, and Pt@Cu/TiO_2_ composites act as electron scavenger centers and own more free electrons for reactions [25–27]. However, the generated electron affinity of metal NPs is sometimes insufficient and cannot prevent recombination or maintain electrons for further reactions.

As an advanced solution for catalysis modification, p–n junction photocatalysts with an intrinsic electric field formed at the interface have emerged, which effectively force charge carriers to move in opposite directions and hinder recombination [31–33]. Very recently, Cu_2_O/BiVO_4_, Ag_2_O/Bi_12_O_17_Cl_2_ and CuFe_2_O_4_/Bi_4_Ti_3_O_12_ composite powders have shown improved efficiencies in water treatment based on p–n configuration advantages [34–36]. However, the wetness impregnation synthesis of those powder co-catalysts faces the issues of low surface area, low reproducibility, and difficult control of large-scale production. Therefore, the development of novel catalysts with unique morphologies by using precise tools is extremely essential and important [37–39].

Herein, we constructed a robust p–n junction catalyst by atomic layer deposition (ALD) of TiO_2_ thin films on a p-type SiNW substrate for enhancing the photocatalytic efficiency in CH_4_ oxidation. Pristine p-Si wafers have limited surface area and are highly susceptible to mechanical failure because of their brittle nature; in contrast, the etched SiNW arrays exhibit superior optical absorption and enhanced surface catalytic reaction properties. The intimate contact between 1D Si NWs and thin TiO_2_ layers reduces the recombination rate of electron–hole pairs. Additionally, TiO_2_/SiNWs offer flexibility, improved bandgap energy, and enhanced light harvesting across a broad spectrum, leading to higher photocatalytic efficiency. Combining SiNWs and TiO_2_ presents an opportunity to leverage the strengths of both materials while mitigating their respective limitations. This study offers new insights into the design of an efficient system for OCM.

## Results and Discussion

### Structural and morphological properties

**Figure 1:**
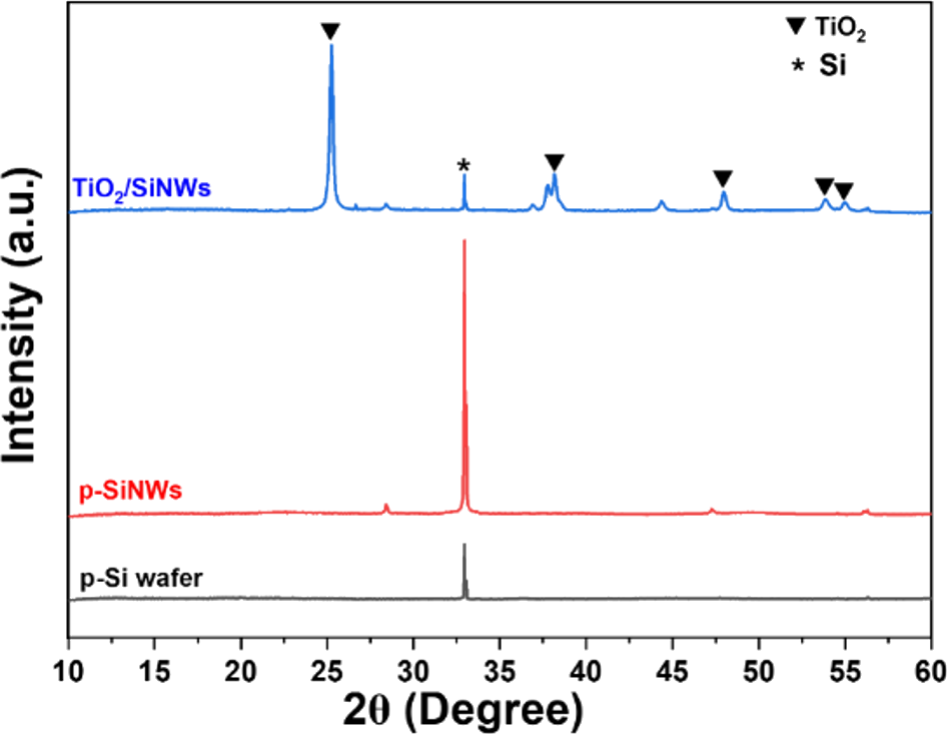
XRD patterns of p-Si, p-type SiNWs, and the TiO_2_/SiNWs sample.

For understanding the crystalline structure of TiO_2_ and SiNWs, X-ray diffraction patterns were recorded as displayed in Figure 1. The XRD pattern of a pure Si wafer and p-type SiNWs display a main peak at 2θ of 33.2°, which was attributed to the reflection from (200) planes. Despite being etched with concentrated acid and Ag^+^ ions, there was no significant change in the peak position of p-type SiNWs, which corresponds to the original Si phase (JCPDS No.27-1402) [40–41]. In the case of the as-synthesized composite, the favored growth of TiO_2_(101) on the surface of p-Si NWs has been noticed [42]. The minor (112), (200), (105), and (211) peaks at 2θ = 38.2°, 48.5°, 53.3°, and 55.1° indicate the formation of anatase TiO_2_ (JCPDS No.21-1272) [43–44]. As expected, the crystal orientation of the TiO_2_/SiNWs catalyst obviously led to the creation of a robust p–n junction photocatalyst.

**Figure 2:**
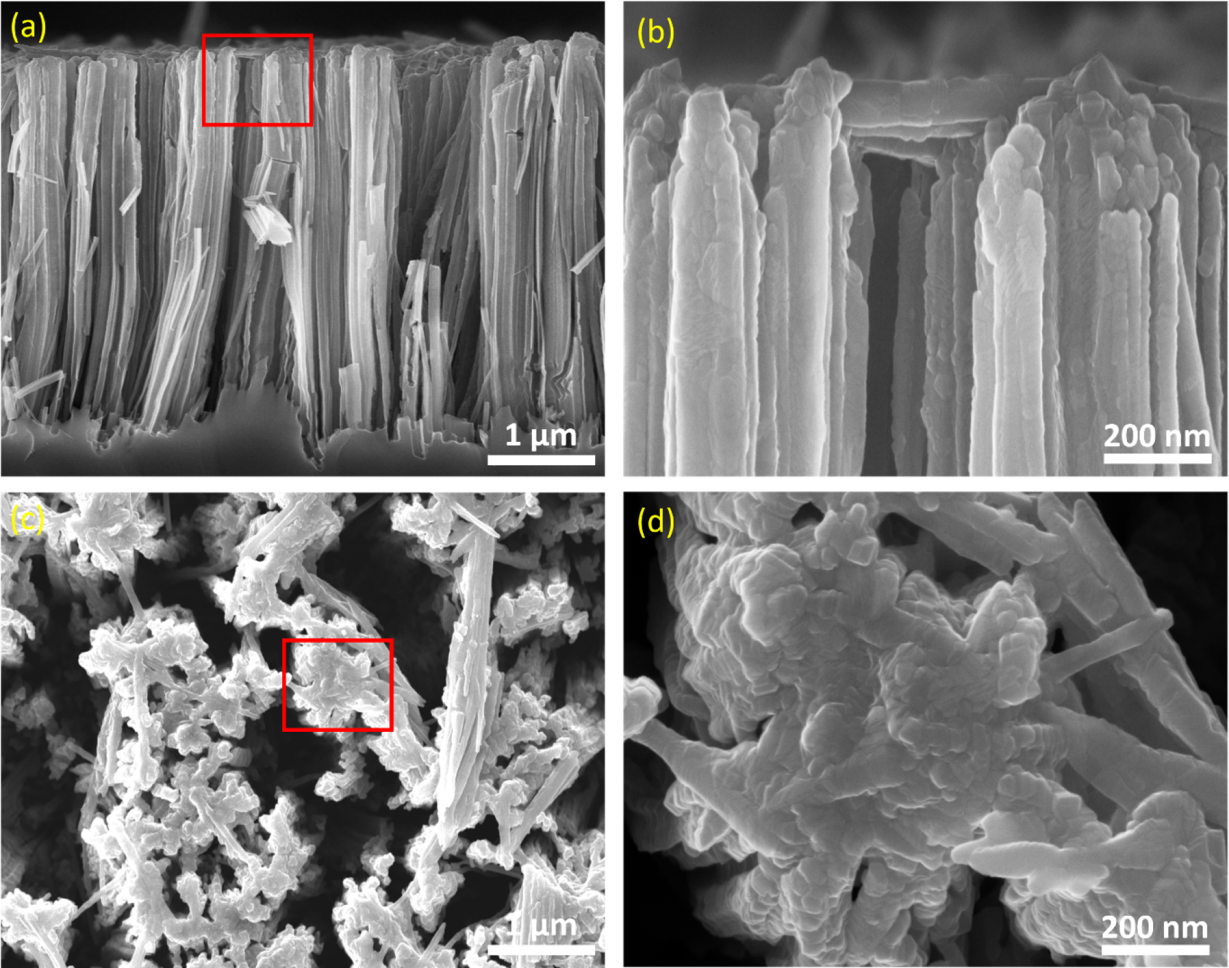
Cross-sectional view (a, b) and top view (c, d) SEM images of the TiO_2_/SiNWs device with a higher-magnification view of the red rectangles on the right-hand side. A thin layer of TiO_2_ (estimated at 25 nm) was deposited on the SiNWs by using ALD with 500 deposition cycles.

The SEM analysis further confirms the morphological evolution during etching and TiO_2_ ALD. As revealed in Supporting Information File 1, Figure S1, the morphology of SiNWs is characterized by individual nanowires that bunch together in a highly ordered manner, resulting in well-oriented Si NW arrays perpendicular to the Si bulk surface. Figure 2 shows cross-sectional- and top-view SEM images of the as-prepared TiO_2_/SiNWs sample. The TiO_2_/SiNWs arrays were well prepared with an average length of 4 µm. Moreover, the surface of the SiNWs was fully decorated by the TiO_2_ passivation layer and became blurry. The active pure 25 nm TiO_2_ layer exhibits flake-like morphology as displayed in Figure 2c,d.

### Optical properties

**Figure 3:**
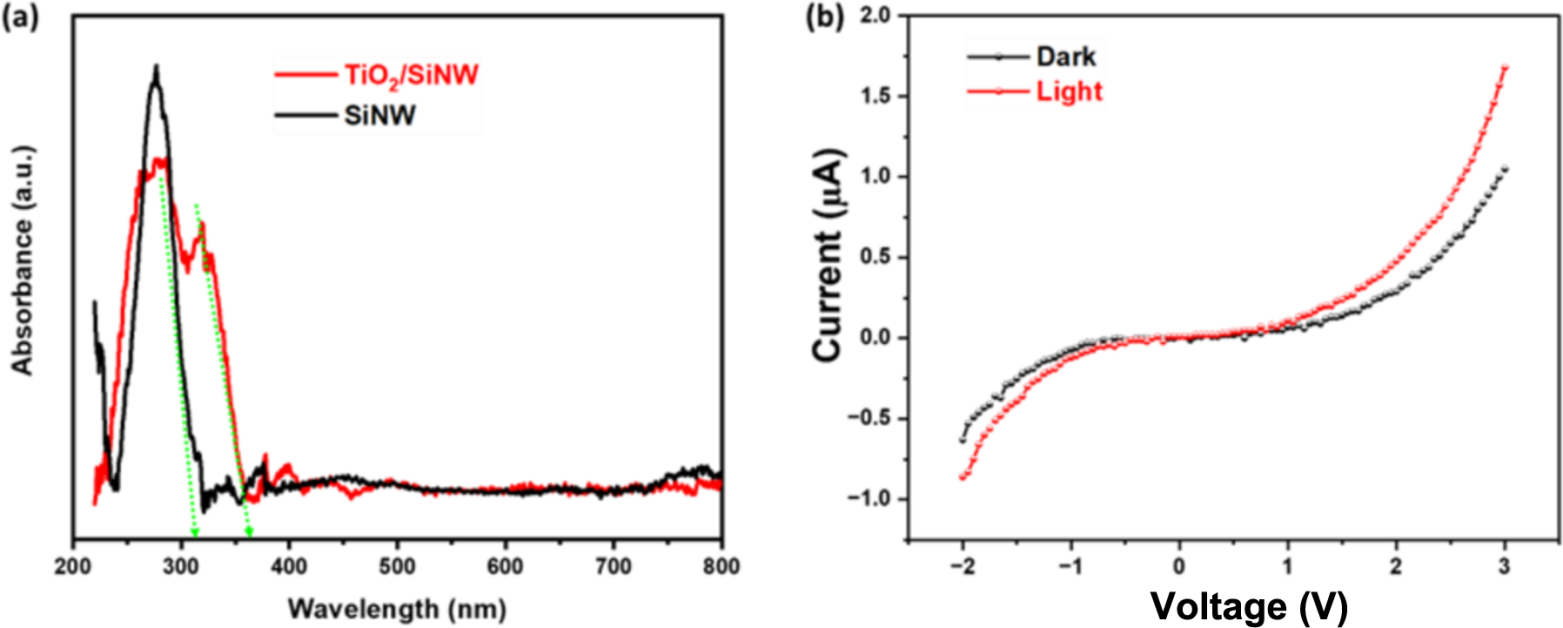
The UV–visible diffuse reflectance spectra (a) and *I*–*V* curve under light/dark conditions (b) of the TiO_2_/SiNWs sample.

Nanowire arrays offer a better optical absorption than planar Si wafers because of the specific morphology [45]. The optical absorption of the as-prepared catalyst is shown in Figure 3a. The UV–vis diffuse reflection spectrum of TiO_2_/SiNWs catalyst is drastically reduced in comparison to the pure SiNWs. The superior antireflection property of the TiO_2_/SiNWs catalyst may be attributed to the vertical wires, which enable strong light scattering leading to enhancement in light harvesting. The optical bandgap values of SiNWs and TiO_2_/SiNWs are estimated at around 3.8 and 3.3 eV, respectively. Figure 3b displays the current–voltage (*I*–*V*) curves of the photocatalyst under dark and light conditions. The current of the sample under light conditions is higher than that under dark conditions. The slope of the *I*–*V* characteristic starts to increase, showing that generated electrons strongly influence the electrical properties of the samples.

The surface interaction with gases during photocatalytic oxidative coupling can be analyzed using water contact angle analysis (as shown in Supporting Information File 1, Figure S2). The wettability of pure p-Si and the p-Si NW array are illustrated in Figure S3 (Supporting Information File 1). Pure p-Si had a water contact angle of 50.24°. Because of the nanowire array morphology, the p-Si NWs were more hydrophilic nature with a water contact angle of 3.36°, which manifests superior photocatalytic oxidative coupling.

**Figure 4:**
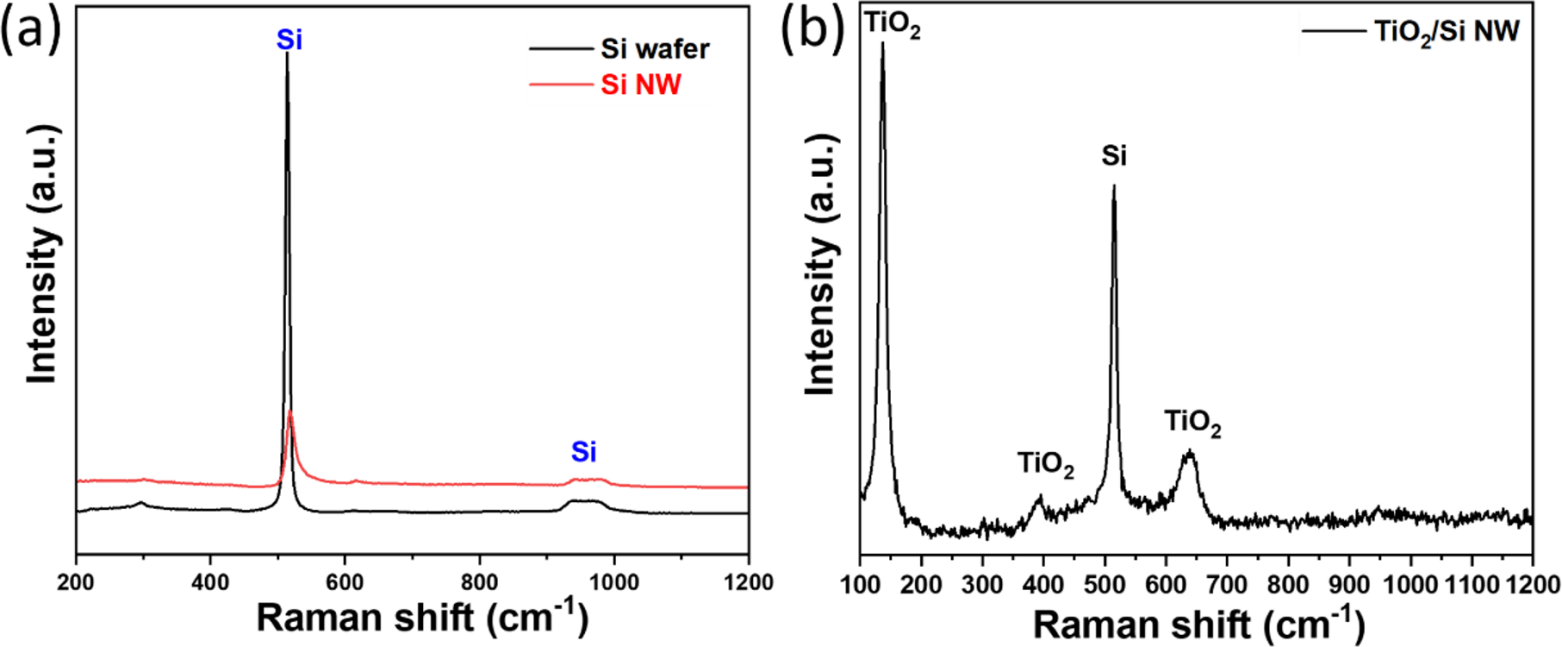
Raman spectra of pure p-Si and Si NW (a), and the TiO_2_/SiNWs thin film (b).

Raman spectra were conducted to confirm the surface composition of the synthesized photocatalysts. As depicted in Figure 4a, the Raman spectrum of Si exhibits a single peak located at 519 cm^−1^, corresponding to the first-order transverse optical (TO) mode of Si [46]. For the TiO_2_/Si photocatalyst, two distinct peaks were observed, namely, (i) the characteristic *E*_g_ vibration of TiO_2_, located at 146 cm^−1^, and (ii) the TO phonon mode of Si (Figure 4b) [47–49]. Consequently, the combined surface-sensitive Raman and bulk-sensitive XRD results reveal that the n-type TiO_2_ coating layer on p-type SiNWs does not influence the crystalline structure.

### Photocatalytic OCM

**Figure 5:**
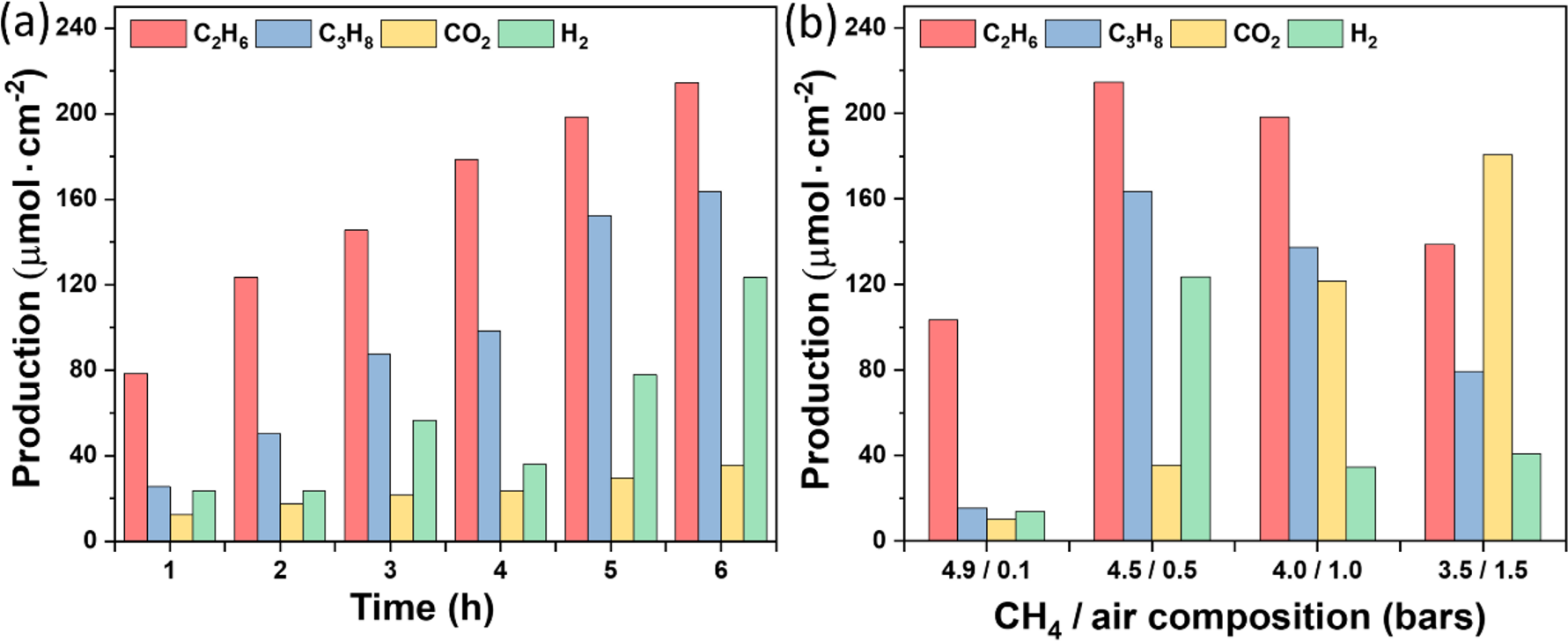
Photocatalytic CH_4_ oxidation as a function of irradiation time (a) and different CH_4_/air composition (b) over TiO_2_/SiNWs. Reaction conditions: TiO_2_/SiNWs chip (1 × 2 cm^2^), total pressure of 5 bar, 20 °C, reaction time *t* = 6 h in (b).

The photocatalytic OCM activity of TiO_2_/SiNWs sample under aerobic conditions is described in Figure 5. In a batch reactor, the photocatalytic CH_4_ oxidation progresses as a function of irradiation time. Besides ethane (C_2_H_6_), carbon dioxide (CO_2_) was detected as a by-product. Moreover, propane (C_3_H_8_) and H_2_ were observed as result of the oxidative cross-coupling of methane and ethane (Figure 5a). The conversion reaction of CH_4_ can be described as follows:




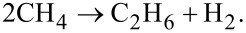




To evaluate the reaction ratio-dependent photocatalytic OCM efficiency, we varied the gas pressure ratios between CH_4_ and air. As shown in Figure 5b, more CO_2_ was measured at lower CH_4_-to-air ratios because of the higher O_2_ content. Therefore, optimizing the CH_4_/air ratio is important for improving the OCM reaction. The highest coupling selectivity was around 90% at CH_4_/air ≈ 4.5:0.5, comparable to or higher than that of typical reported photocatalysts (Table 1). Although the TiO_2_/Si composite did not achieve the best results compared to other photocatalysts, it remains a viable option for methane oxidation processes.

**Table 1:** Comparison of the OCM performance of the TiO_2_/Si composite with previous photocatalysts.

Photocatalysts	Amount of catalyst	Reaction conditions	Light	Productivity (μmol·g^−1^·h^−1^) and selectivity	Ref.

Au@Zn_2_Ti_3_O_8_	0.03 g	CH_4_/O_2_ = 15:1, mild conditions	50 W LED	C_2_H_6_: 1219 Sel.: 81%	[50]
Pt@TiO_2_	0.075 g	CH_4_ and water	UV lamp	C_2_H_6_: 57 Sel.: 62%	[51]
AuZnO@TiO_2_	0.02 g	CH_4_/air = 69:1, mild conditions	300 W Xe lamp	C_2_H_6_: 5020 Sel.: 90%	[30]
Ag HPW@TiO_2_	0.1 g	CH_4_ and air, 0.3 MPa	400 W Xe lamp	C_2_H_6_: 21 Sel.: 90%	[52]
Au@ZnO	0.005 g	CH_4_/O_2_ = 99:1, mild conditions	2 LEDs	C_2_-C_4_:684 Sel.: 83%	[53]
TiO_2_/Si	1 × 2 cm^2^	CH_4_/air = 4.5:0.5, 0.5 MPa	300 W Xe lamp	C_2_H_6_: 210 µmol/cm^2^ in 6 h Sel.: 90%	this work

**Figure 6:**
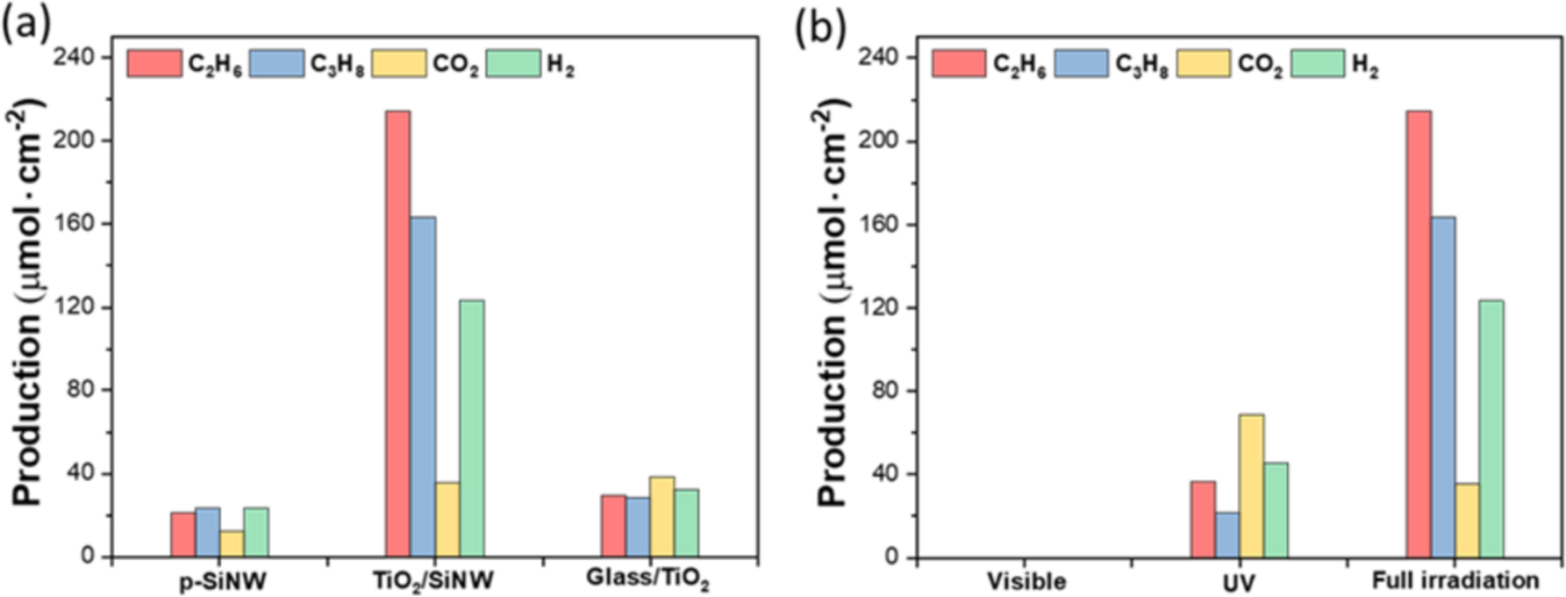
Photocatalytic CH_4_ oxidation over different types of catalyst (a), and under different types of irradiation (b). Note that only TiO_2_/SiNWs was used in panel (b). Reaction conditions: 5 bar, 20 °C, *t* = 6 h.

In order to investigate the individual effects of p-type SiNWs and TiO_2_ layer on the photocatalytic OCM efficiency, comparable samples were irradiated under the same conditions. The methane coupling was analyzed with different samples using pure p-type SiNW, TiO_2_/SiNW, TiO_2_/glass catalyst, as shown in Figure 6a. In fact, only negligible CH_4_ conversion was discovered over p-type SiNWs, and TiO_2_/glass under the same conditions. It can be explained that the superior wettability of TiO_2_/SiNWs (hydrophilic surfaces) compared to TiO_2_/glass (hydrophobic surfaces) enables partial adsorption of water molecules, which facilitates the generation of radicals necessary for photocatalytic reactions. Furthermore, the TiO_2_ nanostructure and p-type SiNWs are crucial in photogenerated charge separation and adsorption enhancement under UV–vis light. In other words, the ethane productivity of TiO_2_/Si NWs was five times higher (210 µmol/cm^2^) than that of pure p-Si NWs (20 µmol/cm^2^) and glass/TiO_2_ (30 µmol/cm^2^).

Photocatalytic OCM over TiO_2_/Si NWs catalysts was recorded in a batch reactor under different wavelengths of light. As shown in Figure 6b, no products were detected under the visible-light irradiation. The photocatalytic performance under UV illumination was significantly lower than that under full illumination.

**Figure 7:**
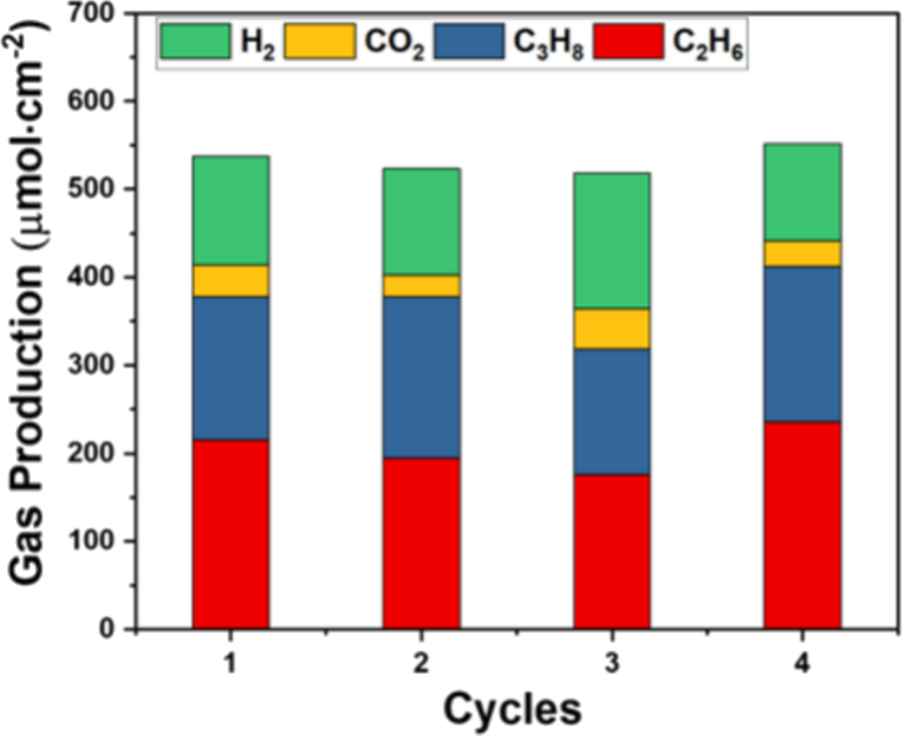
Stability of photocatalytic CH_4_ oxidation reaction over the TiO_2_/SiNWs sample.

Figure 7 shows the recyclability of the p–n TiO_2_/SiNWs photocatalyst. Note that after each cycle, the reactor was completely evacuated, and fresh gases were refilled for the following run. The photocatalytic activity of p–n TiO_2_/SiNWs remained almost unchanged after four consecutive reaction cycles, indicating a high recyclability in the batch reactor.

### Plausible charge transport mechanism

**Figure 8:**
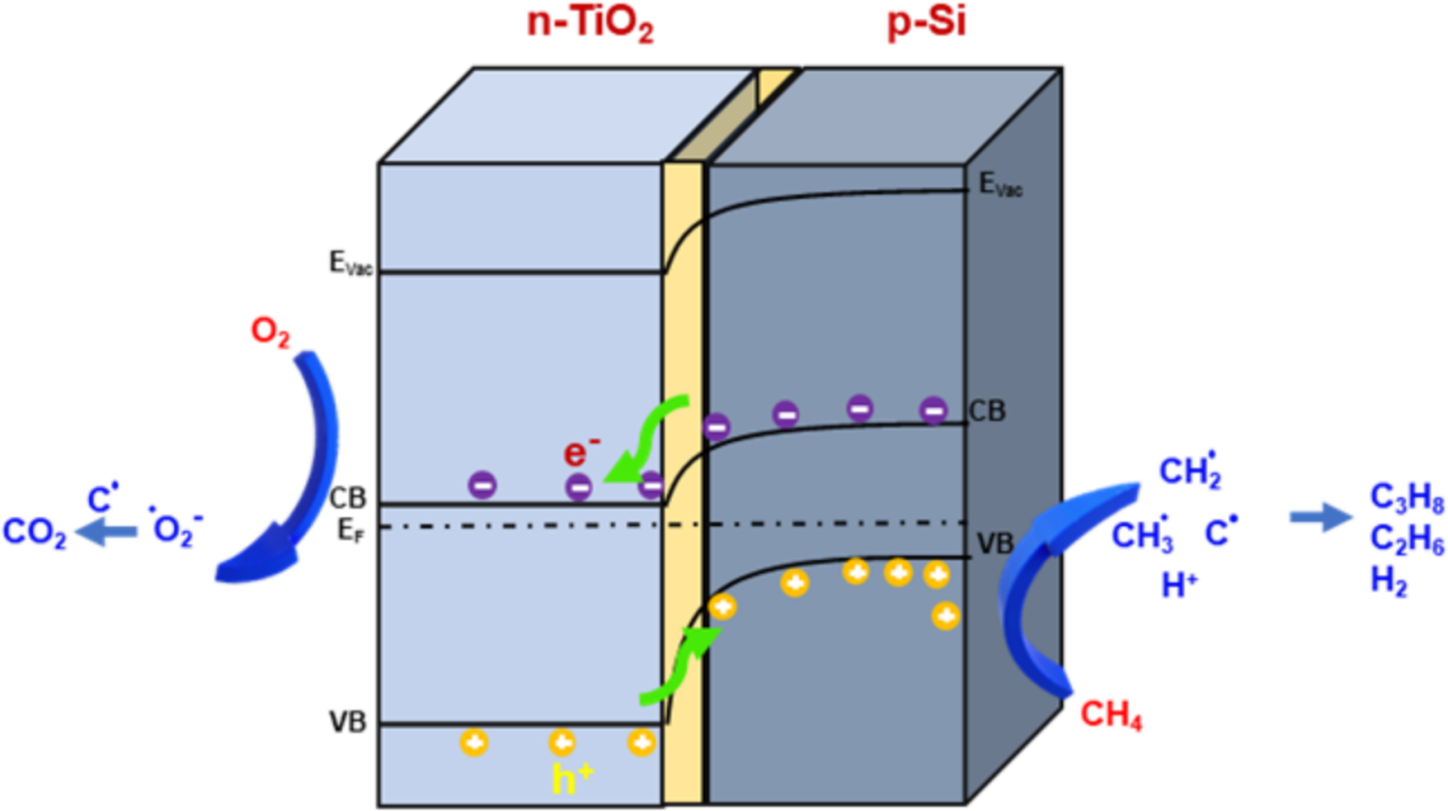
Proposed mechanism of photocatalytic CH_4_ oxidation reaction over p–n junction TiO_2_/SiNWs sample.

Figure 8 shows a schematic mechanism of the photocatalytic activity of the p–n TiO_2_/SiNWs hierarchical structures. According to previous studies the energy bandgaps of p-Si and n-type TiO_2_ were assumed to be 1.1 eV and 3.3 eV, respectively [54–56]. Because of the different electron affinities (*E*_ea,TiO2_ ≈ 4.10 eV, *E*_ea,Si_ ≈ 4.05 eV) [57–58] the electrons are excited and moved from the valance band minimum (VBM, 0.74V vs NHE) of SiNWs to the conduction band maximum (CBM, 0.35V vs NHE) of TiO_2_ to enter the equilibrium state under irradiation following Aderson’s model [39]. The photogenerated electrons tend to produce C_2_H_6_, C_3_H_8_, and H_2_ from H^+^. The photogenerated holes at the VBM of TiO_2_ create oxidized intermediates and H^+^ [59–61]. The whole process can be expressed by the S-scheme mechanism, as follows:




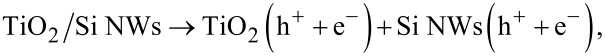




at the CB of n-type TiO_2_




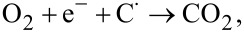




and at the VB of p-Si NW




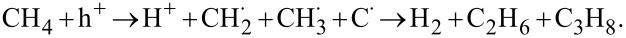




## Conclusion

A p–n junction TiO_2_/SiNWs photocatalyst was synthesized via ALD and utilized for light-driven OCM. TiO_2_/SiNWs revealed excellent performance owing to the smooth transport of photogenerated electrons in the p–n junction, which lowers the e–h recombination rate. The nanowire array structure of the catalyst provides a surface that can massively increase light absorption, achieving an efficient C_2_H_6_ yield of 210 µmol/cm^2^ in 6 h with high selectivity under light illumination at room temperature. This research could offer new insights into composite photocatalysts for methane coupling.

## Experimental

### Chemicals and materials

Commercial p-type Si 3-inch wafers (⟨100⟩ orientation, boron-doped, resistivity = 0.01–1 Ω·cm) were purchased from Silicon Mitus Corporation, South Korea. Silver nitrate (AgNO_3_, 0.1 M), hydrofluoric acid HF (50 wt %), nitric acid (HNO_3_, 63%), acetone, and ethanol were provided from Sigma-Aldrich. Deionized (DI) water was used for cleaning steps.

#### Si NWs and TiO_2_/Si NWs preparation

**Figure 9:**
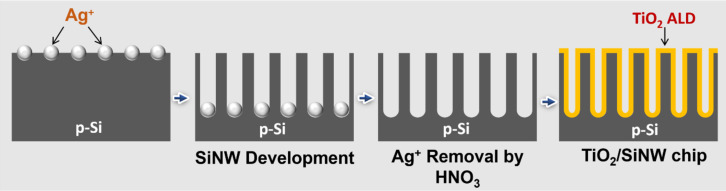
Schematic illustration of TiO_2_/SiNWs chip fabrication starting from a p-type silicon wafer. A thin layer of TiO_2_ was deposited on the Si NWs via ALD.

First, a small piece (1 × 2 cm^2^) was cut from a commercial p-type Si wafer and washed several times using DI water, ethanol, and acetone in a sonication bath. Etching solution containing AgNO_3_ (0.1 M), HF (50 wt %) and H_2_O (2:1:2 vol %) was prepared and kept at 56 °C for 20 min. The clean Si substrate was rapidly immersed in the etching medium and etched by the Ag^+^ ions for 25 min to obtain 4 µm long SiNWs. Afterwards, remaining Ag on the Si surface was removed using HNO_3_ (63 wt %) for 10 min. The etched p-Si NWs substrate was eventually washed with DI water and dried under N_2_ flow, as shown in Figure 9.

Second, the as-prepared SiNWs sample was transferred to the chamber of an ALD system (R200 Advanced Picosun, 2013) for TiO_2_ thin film deposition. The TiO_2_ thin film deposition was carried out at 300 °C using TiCl_4_ (98% purity, 0.2 s pulse time) and H_2_O (0.1 s pulse time) as precursors. The vacuum level of the chamber was kept at 8 × 10^−3^ bar, and the deposition rate was 0.051 nm/cycle. After cooling down to room temperature, the sample was taken out from chamber for photocatalytic experiments.

#### Photocatalytic tests

The as-synthesized catalyst was placed in a custom-made batch reactor with a small transparent quartz window, which was directly connected to a gas chromatograph (GC) with thermal conductivity and flame ionization detectors. A 300 W Xenon lamp was utilized as a light source (HAL-320). First, the reactor containing the photocatalytic thin film samples was evacuated using a vacuum pump for 10 min and filled with a mixture of CH_4_/air (4.5/0.5 pressure ratio). The pressure ratio of the gas mixture was varied to study gas composition-dependent efficiency. The total pressure of gaseous reactants in batch reactor was established at 5 bar, and the photocatalytic system was kept in the dark (20 min) to reach equilibrium before exposure to simulated solar light. The temperature was kept at room temperature via a cooler.

#### Characterization

The crystalline structure and morphological properties of as-synthesized samples were analyzed using an X-ray diffraction system (XRD, Rigaku, SmartLab) with a 2θ range of 20–80° and a field-emission scanning electron microscope (FE-SEM, Hitachi, S-4700). The absorption properties of the thin films were analyzed using a diffuse reflectance UV–vis spectrometer (DRS-UV, Shimazu UV-2450). The chemical structure of the catalyst surface was analyzed using a Raman spectrometer (excitation of 532 nm, ANDOR Monora 500i). The surface wettability of the thin film sample was measured using a static contact angle system (Biosin Scientific), as shown in Supporting Information File 1, Figure S2. The contact angle between horizontal sample surface and the perimeter of the water drop was measured after 10 s of interaction. The in situ photocurrent measurements were carried out in the presence of gaseous reactants (CH_4_/air = 4.5/0.5) ranging from −2 V to +3 V under dark and light conditions. Each measurement was scanned with 0.05 V intervals. The bias supply and current signals were provided and recorded by a Keithley system adapted with an amplifier.

## Supporting Information

File 1 Additional figures.

## Data Availability

The data that supports the findings of this study is available from the corresponding author upon reasonable request.
