# Supplementary material for: Photocatalytic methane oxidation over a TiO2/SiNWs p–n junction catalyst at room temperature
Source: Beilstein J Nanotechnol. 2024 Sep 2;15:1132–41. doi: 10.3762/bjnano.15.92 (PMC11403797; doi:10.3762/bjnano.15.92)
Supplement: File 1 — Additional figures. [file Beilstein_J_Nanotechnol-15-1132-s001.pdf]

BEILSTEIN JOURNAL OF NANOTECHNOLOGY

Supporting Information

for

Photocatalytic methane oxidation over a TiO_2/SiNWs p–n junction catalyst at room temperature

Qui Thanh Hoai Ta, Luan Minh Nguyen, Ngoc Hoi Nguyen, Phan Khanh Thinh Nguyen and Dai Hai Nguyen

Beilstein J. Nanotechnol. **2024,** *15,* 1132–1141. doi:10.3762/bjnano.15.92

Additional figures

License and Terms: This is a supporting information file under the terms of the Creative Commons Attribution License (https://creativecommons.org/licenses/by/4.0). Please note that the reuse, redistribution and reproduction in particular requires that the author(s) and source are credited and that individual graphics may be subject to special legal provisions.
The license is subject to the *Beilstein Journal of Nanotechnology* terms and conditions: (https://www.beilstein-journals.org/bjnano/terms)

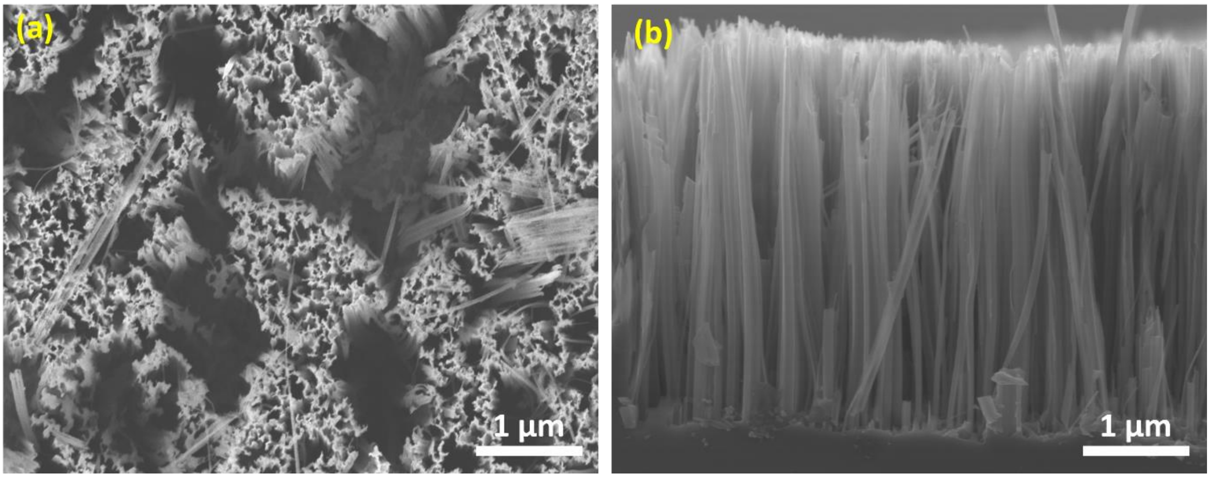

Figure S1: SEM images of SiNWs after etching under top view (a) and cross-sectional view (b).

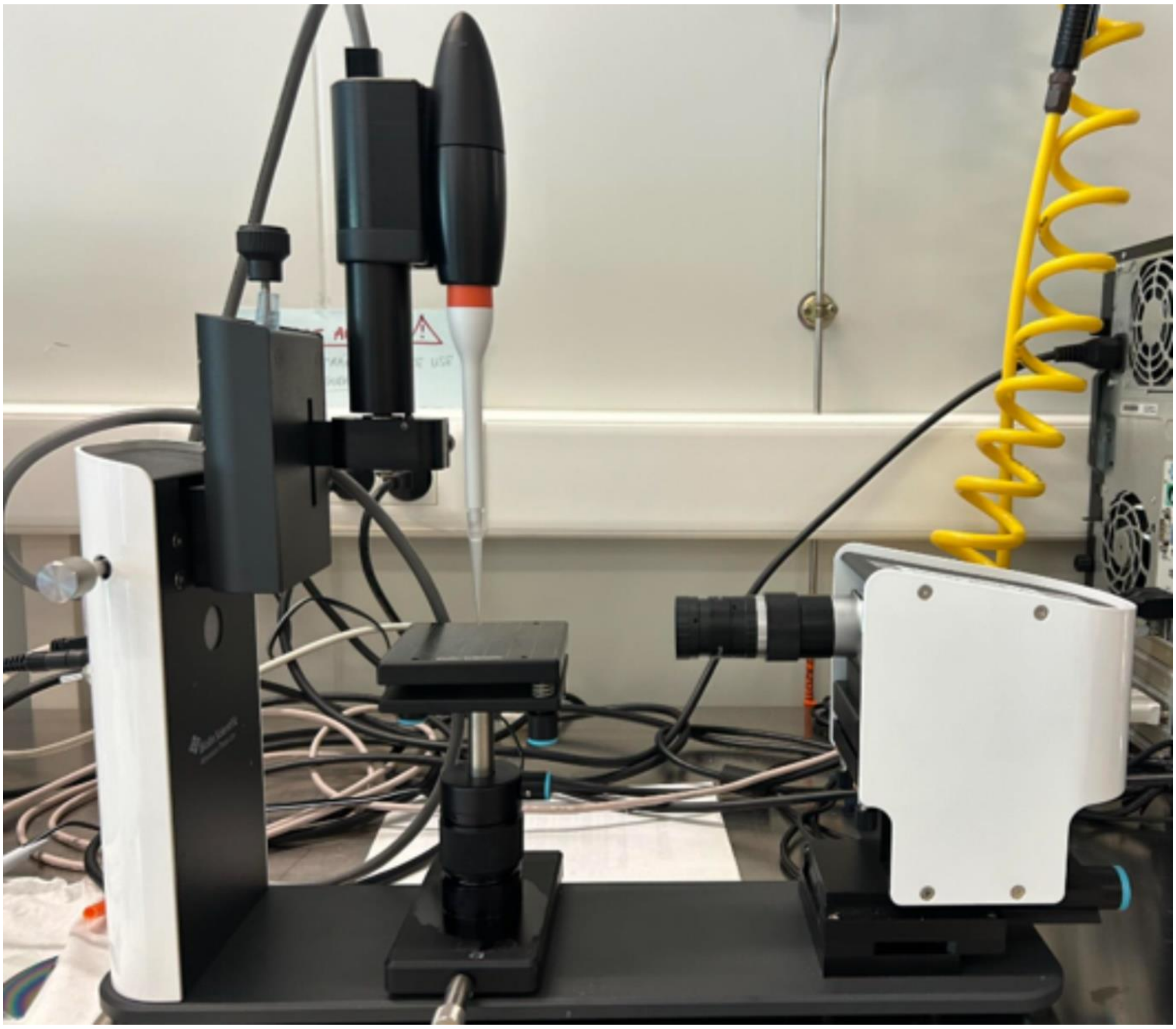

Figure S2: Contact angles measurment set up (Biosin Scientific). The thin film samples were placed on specimen substrate and the sample-water droplet interaction was recorded by an optical camera.

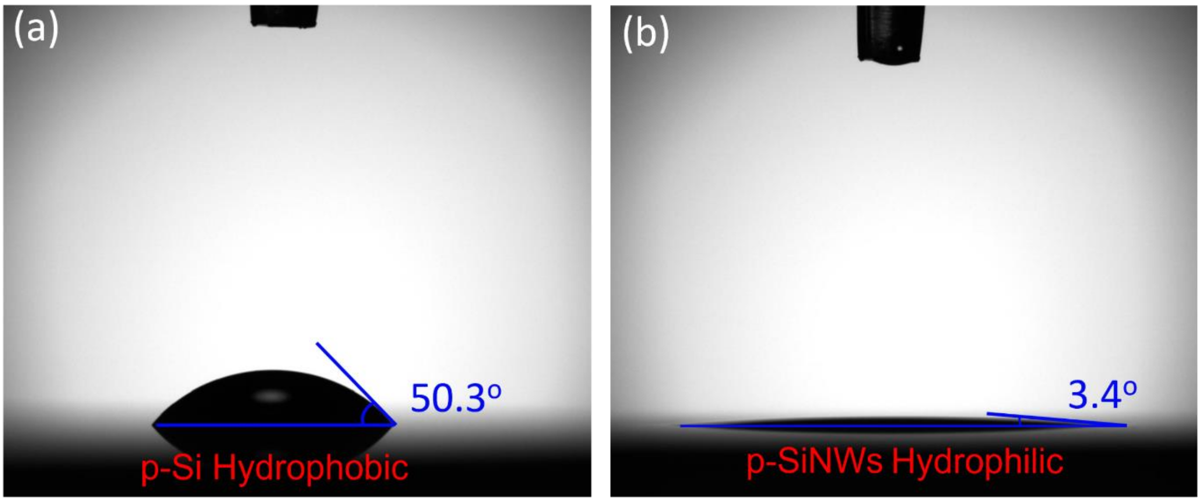

Figure S3: Contact angles of (a) p-Si and (b) p-SiNWs samples.
